# Biomechanical effects of interbody cage height on adjacent segments in patients with lumbar degeneration: a 3D finite element study

**DOI:** 10.1186/s13018-022-03220-3

**Published:** 2022-06-21

**Authors:** Xiao Lu, Dachuan Li, Hongli Wang, Xinlei Xia, Xiaosheng Ma, Feizhou Lv, Fei Zou, Jianyuan Jiang

**Affiliations:** Department of Orthopedics, Huashan Hospital, Fudan University, No. 12, Middle Wulumuqi Road, Jing’an District, Shanghai, 200040 China

**Keywords:** TLIF, Interbody cage, Intervertebral height, Degenerative lumbar spine, Biomechanics

## Abstract

**Objective:**

To investigate the biomechanical effects of interbody cage height on adjacent segments in patients with lumbar degeneration undergoing transforaminal lumbar interbody fusion (TLIF) surgery, so as to provide references for selection of interbody cage.

**Methods:**

The finite element model of normal lower lumbar spine (L3–S1) was built and validated, then constructed three different degenerative segments in L3–L4, and the cages with different height (8, 10, 12, 14 mm) were implanted into L4–L5 disc. All the twelve models were loaded with pure moment of 7.5 N m to produce flexion, extension, lateral bending and axial rotation motions on lumbar spine, and the effects of cage height on range of motion (RoM) and intervertebral pressure in lumbar spine were investigated.

**Results:**

The RoM of adjacent segments and the maximum stress of intervertebral discs increased with the increase in cage height, but this trend was not obvious in mild and moderate degeneration groups. After implantation of four different height cages (8, 10, 12, 14 mm), the RoM of L3/L4 segment reached the maximum during extension. The RoM of mild degeneration group was 2.07°, 2.45°, 2.48°, 2.54°, that of moderate degeneration group was 1.79°, 1.97°, 2.05°, 2.05°, and that of severe degeneration group was 1.43°, 1.66°, 1.74°, 1.74°. The stress of L3–L4 intervertebral disc reached the maximum during flexion. The maximum stress of L3–L4 intervertebral disc was 20.16 MPa, 20.28 MPa, 20.31 MPa and 20.33 MPa in the mild group, 20.58 MPa, 20.66 MPa, 20.71 MPa and 20.75 MPa in the moderate group, and 21.27 MPa, 21.40 MPa, 21.50 MPa and 21.60 MPa in the severe group.

**Conclusion:**

For patients with mild-to-moderate lumbar degenerative disease who need to undergo TLIF surgery, it is recommended that the height of fusion cage should not exceed the original intervertebral space height by 2 mm, while for patients with severe degeneration, a fusion cage close to the original intervertebral height should be selected as far as possible, and the intervertebral space should not be overstretched.

## Introduction

Lumbar disc degeneration is a progressive disease that causes alterations in the geometric morphology and biomechanical properties of lumbar discs, ultimately affecting the transmission and allocation of human gravity by lumbar spine [1]. The causes of lumbar disc degeneration are complex and diverse, including ageing, abnormal mechanical loading, trauma and so on [2–4]. Among the causes of low back pain, lumbar disc degeneration accounts for up to 40% [5, 6], and this number is climbing year by year [7, 8], resulting in a serious personal and socio-economic burden.

Transforaminal lumbar interbody fusion (TLIF) is a classic and widely used lumbar fusion surgery, and this method is transforaminally implanted with interbody fusion devices (cage), which preserves the integrity of posterior structures such as the contralateral lamina to a greater extent, thereby reducing the traction effect on nerve roots and dura mater and reducing the possibility of nerve injury. Because TLIF excises only one zygapophyseal joint and has many important advantages such as less effect on spinal stability, avoidance of nerve injury, higher fusion rate and fewer complications, it has been widely used in the clinic [9–11].

Adjacent segment degeneration (ASD) is a common complication after lumbar interbody fusion, and symptomatic ASD can negatively affect the effect of surgery and lead to further surgery and higher medical costs, so it receives increasing attention from clinicians and researchers [12]. Based on radiographic evidence, the prevalence of ASD is reported to be more than 40%, and the incidence of symptomatic ASD that requires revision surgery reportedly ranges from 5.2 to 18.5% [13, 14]. Many factors are considered to be related to the development of ASD, such as high body mass index, paraspinal muscle atrophy, interbody fusion and so on [15, 16]. In addition to the above risk factors, the height of interbody fusion cage placed during operation cannot be ignored [17]. One study that focussed on the degree of disc height distraction during surgery suggested that the excessive distraction of the L4–L5 disc space during surgery is a significant risk factor for the development of ASD [18]. However, there is a lack of relevant biomechanical research.

In this paper, a normal lumbar finite element model (L3–S1) was established, and on this basis, three different degrees of degenerative lumbar models (L3–L4) were constructed. Then, TLIF surgery was simulated at L4–L5 segments, and cages with different heights of 8, 10, 12 and 14 mm were implanted. We aimed to analyse the biomechanical effects of cage height on different degrees of degenerative lumbar spine.

## Materials and methods

### Establishment of normal lumbar spine model

**Fig. 1:**
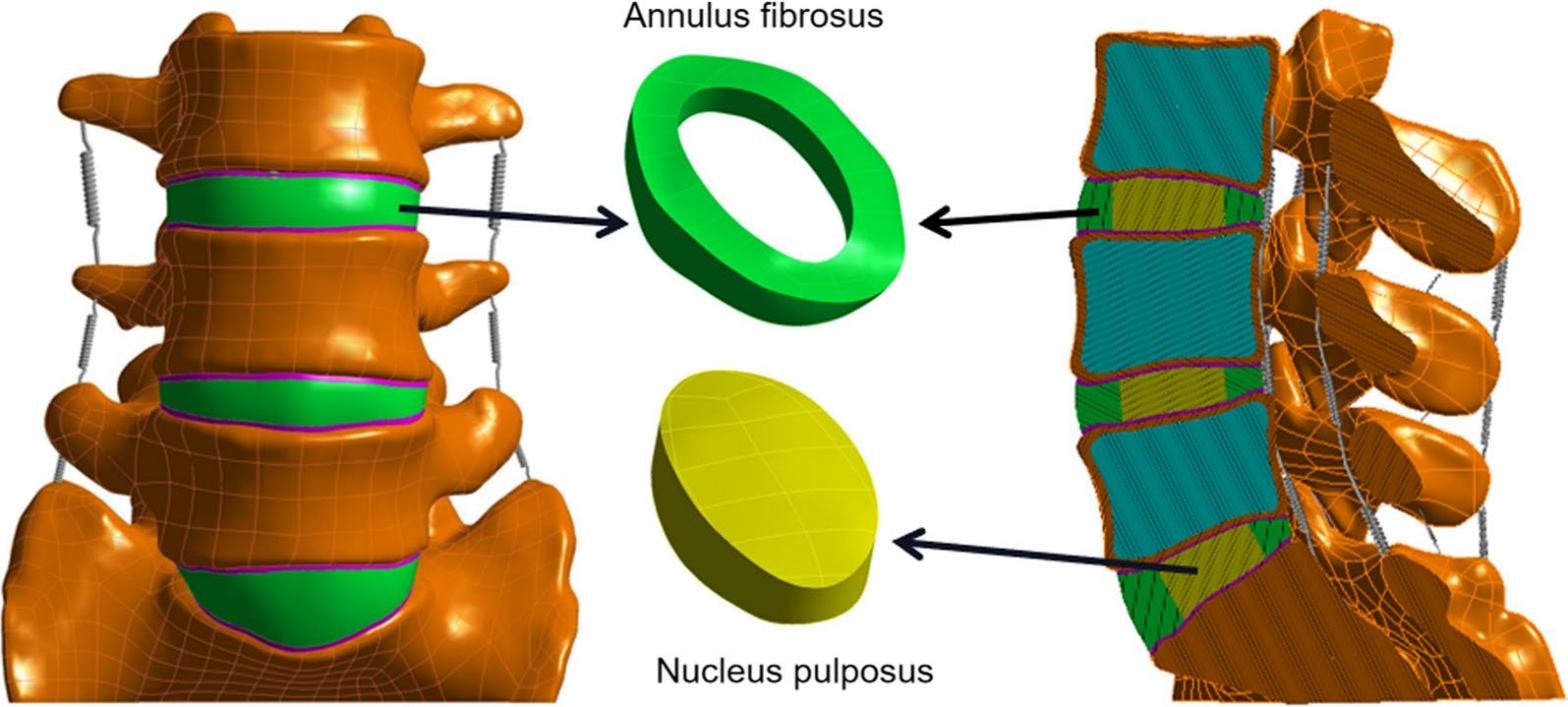
Finite element model of normal lower lumbar spine and intervertebral disc structure

Based on the CT scanning data of a healthy male volunteer (age 24 years, height 170 cm, weight 60 kg), our team established a three-dimensional nonlinear lumbar finite element model of the whole segment of L1–S1. Firstly, CT data were imported into Mimics 21.0 software (Materialise Inc., Belgium), and the geometric contour of L1–S1 segment vertebral body was extracted by using image threshold segmentation, filling, erasing and other functions. The files were then imported into Geomagic 2017 (Geomagic Inc., USA) for surface construction, patching, grinding, denoising, cutting, slicing and smoothing so that the external shape was close to the bony structure of the lumbar spine. The structures of upper and lower endplates, intervertebral discs and articular cartilage were established in SolidWorks 2021 (Dassault Systems Inc., France). In this study, the thickness of cortical bone was set as 1 mm, the upper and lower endplates were seamlessly connected with the upper and lower surfaces of vertebral body, and the thickness of endplate was 0.5 mm. The intervertebral disc was divided into nucleus pulposus and annulus fibrosus, and the nucleus pulposus accounted for 40% of the intervertebral volume [19, 20]. Finally, the vertebral bodies, discs and articular cartilage were imported into the ANSYS Workbench 2021 (ANSYS Inc., USA) for material assignment and assembling, and ligament reconstruction (posterior longitudinal ligament, supraspinous ligament, interspinous ligament, transverse interspinous ligament, joint capsule ligament, ligamentum flavum and anterior longitudinal ligament) was performed by spring unit [21]. The material parameters used in the modelling process are shown in Table 1 [22, 23]. The L3–S1 segment was kept as the study model according to the purpose of the study, as shown in Fig. 1.

**Table 1:** Material properties used in the finite element model

Materials	Young’s modulus	Poisson’s ratio
Cortical bone	12,000	0.3
Cancellous bone	100	0.3
Endplate	24	0.4
Annulus fibrosus	4.2	0.45
Nucleus pulposus	0.1	0.49
Cage	3600	0.25
Anterior longitudinal ligament	7.8	0.3
Posterior longitudinal ligament	10	0.3
Flaval ligament	15	0.3
Transverse ligament	10	0.3
Capsular ligament	7.5	0.3
Interspinous ligament	8	0.3
Supraspinous ligament	8	0.3

### Establishment of lumbar spine models with different degrees of degeneration

**Fig. 2:**
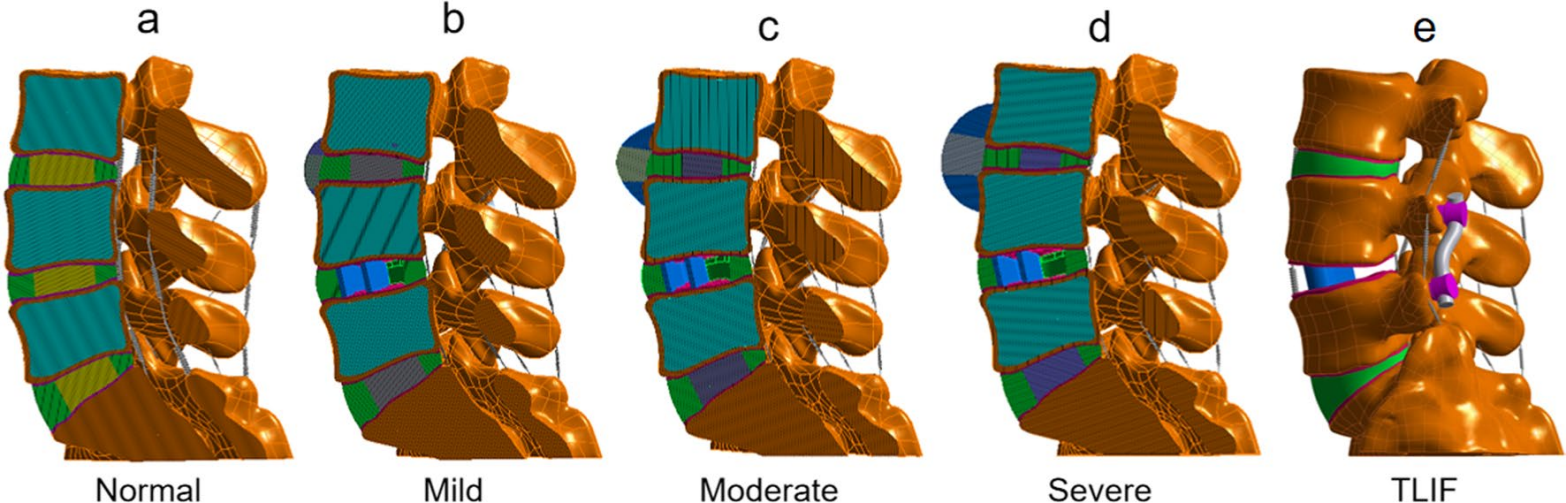
Normal lower lumbar spine model, three different degrees of degenerative lumbar spine models and TLIF operation model. **a** Normal lower lumbar spine model. **b** Mild degeneration model of L3–L4 segment. **c** Moderate degeneration model of L3–L4 segment. **d** Severe degeneration model of L3–L4 segment. **e** TLIF model of L4–L5 segment

Based on the normal lumbar spine model, three lumbar spine models with different degrees of degeneration were constructed by modifying the morphology of lumbar spine (Table 2) and changing the material properties of tissue (Fig. 2). These changes mimic the natural degenerative process in the lumbar spine, including decreased disc height, osteophyte formation and decreased nucleus pulposus area [22, 24]. Since L4–L5 is a common segment in surgery, we chose to construct a degeneration model in L3–L4 segment.

**Table 2:** Changes in lumbar intervertebral disc geometric morphology during disc degeneration

Structure	Normal (%)	Mild (%)	Moderate (%)	Severe (%)
Disc height^a^	100	80	60	40
Height of osteophytes^b^	–	10	20	30
Nucleus pulposus area^c^	100	75	50	40

^a^Intervertebral disc height: The L3 vertebrae moved down 20% (mild), 40% (moderate) and 60% (severe) of the original intervertebral height, respectively

^b^Osteophyte: The height and length of the osteophyte are equal in the sagittal plane. The height and length of the anterior osteophytes were 10% (mild), 20% (moderate) and 30% (severe) of the normal sagittal diameter of L3 and L4, respectively

^c^The surface area of nucleus pulposus: Decreased to 75% (mild), 50% (moderate) and 40% (moderate) of the normal value, respectively, and the reduced part was replaced by annulus fibrosus

**Fig. 3:**
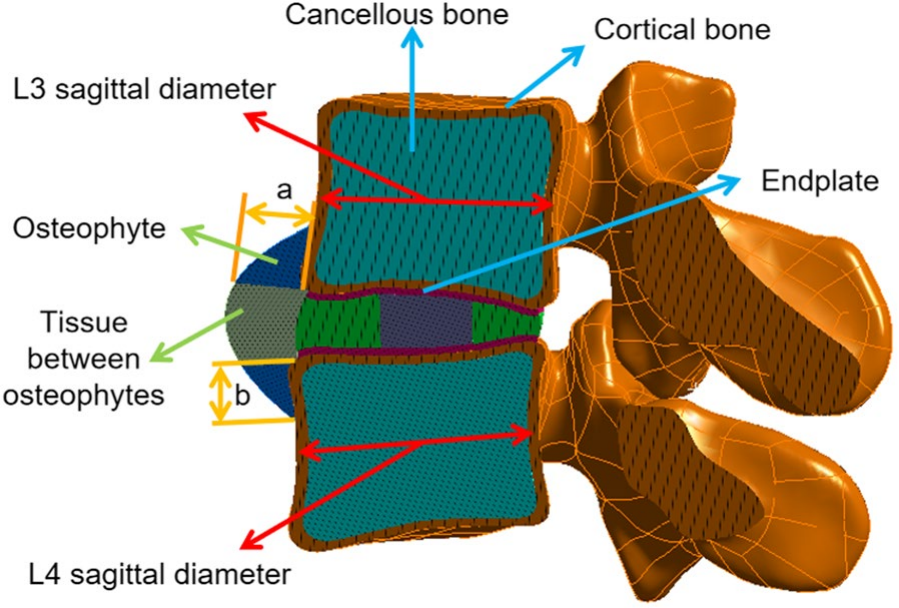
Schematic illustration of osteophyte dimensions

In the normal model, L3 vertebrae were totally displaced by 20% (mild), 40% (moderate) and 60% (severe) of the original intervertebral height [25]. The osteophyte architecture was irregular, and the height and length of the osteophytes were set to be equal in the sagittal plane in order to simplify the model for subsequent manipulation (Fig. 3). The height and length of anterior osteophyte of lower L3 and upper L4 vertebral bodies were 10% (mild), 20% (moderate) and 30% (severe) of the sagittal diameter of normal L3 and L4 vertebral bodies, respectively. According to previous studies [22, 24], the osteophyte and soft tissue components between the upper and lower osteophytes were defined as the components of cancellous bone and annulus fibrosus, respectively. In the process of intervertebral disc degeneration, the volume of nucleus pulposus decreased continuously, and the surface area decreased to about 75% (mild), 50% (moderate) and 40% (severe) of the normal value, respectively. The reduced part was replaced by annulus fibrosus [26].

### Surgical simulation and implantation of cage

Following the clinical operation process, the TLIF operation was simulated at L4–L5 segments to complete the construction of the operation model. Firstly, on the basis of three different degrees of L3–L4 degeneration models, the right facet joint was removed, then the whole nucleus pulposus and some annulus fibrosus of L4–L5 were removed, and the cage was introduced into the defective intervertebral disc. Finally, pedicle screws were placed on both sides for fixation. The contact between the cage and the upper and lower endplates was defined as binding constraint. The cartilage was bound to the corresponding articular process. The friction coefficient between the articular surfaces of the cartilage was 0.2, and the contact between the components was set according to actual conditions [23]. The length and width of the fusion cage were 45 mm and 22 mm, respectively, the length of pedicle screw was 40 mm, the diameter was 6.5 mm, and the length of connecting rod was 53 mm, the diameter was 5.5 mm.

**Fig. 4:**
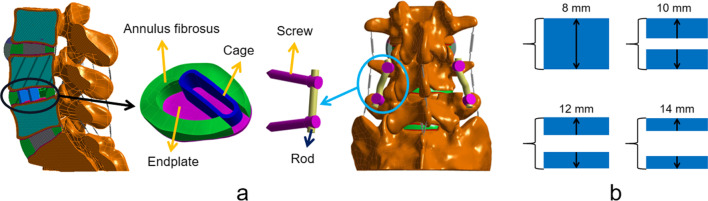
Finite element model of L4–L5 segment TLIF. **a** Schematic diagram of cage. **b** Four different height cages

Since the original intervertebral space height of L4–L5 segments in this study was greater than 7 mm but slightly less than 8 mm, 8 mm was selected as the initial height of the cage. Firstly, the 8-mm-high cage was placed in L4–L5 segments according to the above operation process in SolidWorks 2021, and then the cage was Boolean-operated with L4 and L5 vertebral bodies to cut off a small part of the lower edge of L4 endplate and the upper edge of L5 endplate, so that the intervertebral space height just reached 8 mm (i.e., the original intervertebral space height), so as to build a fully fitted endplate–cage interface. Finally, the cage was cut from the middle in the horizontal direction and divided into upper and lower parts. The upper part could be translated upward by using the displacement load, so as to establish four cage models with different heights (8, 10, 12 and 14 mm) [27] (Fig. 4).

### Load and boundary conditions

We fixed the bottom surface of S1 vertebral body and applied a load of 500 N vertical stress and 7.5 N m torque to the upper surface of L3 vertebral body [28, 29]. Then, in the finite element analysis software ANSYS Workbench 2021, the range of motion (RoM) of different lumbar segments and the maximum von Mises stress of intervertebral disc were calculated under six working conditions of flexion, extension, left bending, right bending, left rotation and right rotation.

## Results

### Verifying the effectiveness of the model

Under the same load and boundary conditions, the ROM of L3/4 and L4/5 measured by the normal model in this study under six different working conditions was compared with the finite element model of Hao et al. [30] and the cadaver research report of Shim et al. [31]. The experimental results of this study were similar to the literature studies (Table 3), which proved that the model was effective and could be used for subsequent research.

**Table 3:** Model validation results

Motion state	L3/4 RoM			L4/5 RoM		
	Model of this study	Hao et al.	Shim et al.	Model of this study	Hao et al.	Shim et al.
Flexion	4.21	4.90	4.36 ± 0.78	4.91	6.10	5.48 ± 0.88
Extension	2.97	2.70	2.97 ± 0.37	2.61	3.90	2.79 ± 0.42
Left lateral	1.19	2.10	1.76 ± 0.72	1.27	2.60	2.23 ± 1.01
Right lateral	1.31	2.30	1.76 ± 0.72	1.27	2.80	2.23 ± 1.01
Left rotation	1.23	1.20	1.45 ± 0.58	1.43	2.30	1.90 ± 0.99
Right rotation	1.19	1.60	1.45 ± 0.58	1.04	2.10	1.90 ± 0.99

### Influence of different height cages on the RoM of adjacent segments

**Fig. 5:**
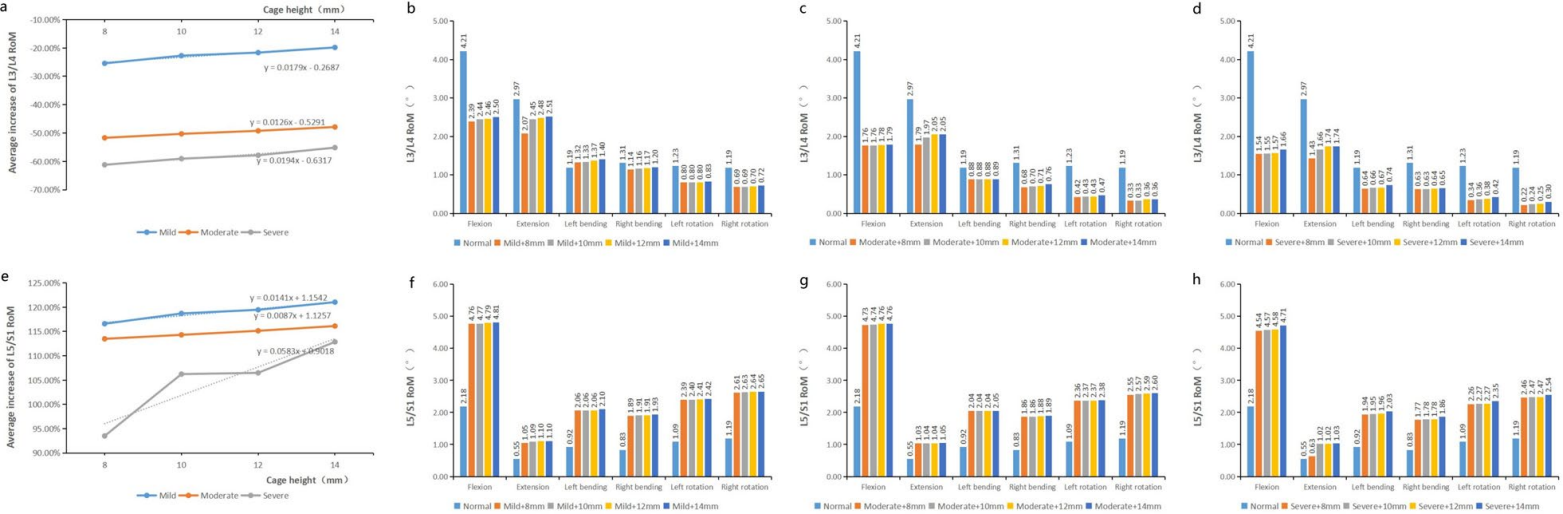
The ROM of adjacent segments. **a** The average increase in L3/4 RoM was linearly correlated with the height of the cage. **b** L3/4 RoM in mild degeneration group. **c** L3/4 RoM in moderate degeneration group. **d** L3/4 RoM in severe degeneration group. **e** The average increase in L5/S1 RoM was linearly correlated with the height of the cage. **f** L5/S1 RoM in mild degeneration group. **g** L5/S1 RoM in moderate degeneration group. **h** L5/S1 RoM in severe degeneration group

In the same degree degeneration models, the RoM of adjacent segments under six working conditions increased with the increase in cage height (Fig. 5). In the mild degeneration group, when four different height cages (8, 10, 12 and 14 mm) were implanted, the RoM reached its maximum in the L3/L4 segment during the extension, which was 2.07°, 2.45°, 2.48° and 2.54°, respectively, and increased by − 30.30%, − 17.51%, − 16.50% and 15.49% compared with the normal model. The RoM of segment L3/L4 in the moderate degeneration group during extension was 1.79°, 1.97°, 2.05° and 2.05°, which was increased by − 39.73%, − 33.67%, − 30.98% and − 30.98% compared with the normal model, respectively. In the severe degeneration group, the RoM of L3/L4 segment during extension was 1.43°, 1.66°, 1.74° and 1.74°, which increased by − 51.85%, − 44.11%, − 41.41% and − 41.41% compared with the normal model, respectively.

For the lower adjacent segment L5/S1, the RoM reached the maximum during flexion in the mild degeneration group. In the four different height cage models, it was 4.76°, 4.77°, 4.79° and 4.81°, respectively, which increased by 118.35%, 118.81%, 119.72% and 120.64%, respectively, compared with the normal model. The RoM of segment L5/S1 in the moderate degeneration group during flexion was 4.73°, 4.74°, 4.76° and 4.76°, which was increased by 116.97%, − 117.43%, 118.35% and 118.35% compared with the normal model, respectively. In the severe degeneration group, the RoM of L5/S1 segment during flexion was 4.54°, 4.57°, 4.58° and 4.71°, which increased by 108.26%, 109.63%, 110.09% and 116.06% compared with the normal model, respectively.

### Influence of different height cages on the maximum stress of adjacent segments

**Fig. 6:**
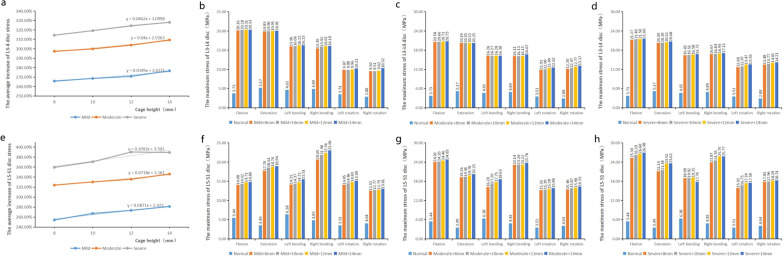
The maximum stress of adjacent intervertebral discs. **a** The average increase in L3–L4 stress was linearly correlated with the height of the cage. **b** L3–L4 stress in mild degeneration group. **c** L3–L4 stress in moderate degeneration group. **d** L3–L4 stress in severe degeneration group. **e** The average increase in L5–S1 stress was linearly correlated with the height of the cage. **f** L5–S1 stress in mild degeneration group. **g** L5–S1 stress in moderate degeneration group. **h** L5–S1 stress in severe degeneration group

In the same degree degeneration model, with the increase in cage height, the maximum stress of adjacent intervertebral discs under six working conditions increased (Fig. 6). In the mild degeneration group, four cages with different heights (8, 10, 12 and 14 mm) were implanted. The stress of L3–L4 intervertebral discs reached the maximum at flexion, which was 20.16 MPa, 20.28 MPa, 20.31 MPa and 20.33 MPa, respectively, increased by 440.48%, 443.70%, 444.50% and 445.04%, respectively, compared with the normal model. The stress of L3–L4 intervertebral disc in the moderate degeneration group during flexion was 20.58 MPa, 20.66 MPa, 20.71 MPa and 20.75 MPa, respectively, which was 451.74%, 453.89%, 455.23% and 456.30% higher than that in normal model. In the severe degeneration group, the stress of L3–L4 intervertebral disc during flexion was 21.27 MPa, 21.40 MPa, 21.50 MPa and 21.60 MPa, respectively, which increased by 470.24%, 473.73%, 476.41% and 479.09%, respectively, compared with the normal model.

For the lower adjacent segment L5–S1 intervertebral disc, the stress reached the maximum during flexion in the mild degeneration group. In the four different height cage models, it was 14.08 MPa, 14.32 MPa, 14.71 MPa and 14.80 MPa, respectively, which was 158.82%, 163.24%, 170.40% and 172.06% higher than that in the normal model. The stress of L5–S1 intervertebral disc in moderate degeneration group was 24.02 MPa 24.20 MPa, 24.46 MPa and 24.83 MPa, which increased by 341.54%, 344.85%, 349.63% and 356.43%, respectively, compared with the normal model. The stress of L5–S1 intervertebral disc in severe degeneration group was 25.30 MPa, 26.20 MPa, 26.60 MPa and 26.98 MPa, respectively, which was 365.07%, 381.62%, 388.97% and 395.96% higher than that in normal model.

## Discussion

The lumbar intervertebral disc is composed of annulus fibrosus, nucleus pulposus and endplate. With the increase in age, the water content of nucleus pulposus decreases, resulting in the narrowing of intervertebral space. The annulus fibrosus also gradually relaxes, and eventually intervertebral disc herniation or even nucleus pulposus prolapse may occur, compressing the nerve root or spinal cord, resulting in corresponding symptoms and signs. TLIF is a classic surgical method for the treatment of lumbar degenerative diseases. It has the advantages of definite therapeutic effect, stable surgical segments and high post-operative fusion rate [10]. The use of appropriate cage during surgery can effectively restore the height of degenerative intervertebral space, but the research on the effect of different height cage on degenerative lumbar spine has not been reported.

In this paper, the degeneration models of mild, moderate and severe degree were established in L3–L4 segment by finite element method, and the biomechanical effects of different height cages (8, 10, 12, 14 mm) implanted in L4–L5 segment on lumbar spine were analysed. Twelve models were loaded with 7.5 N m torque to simulate flexion, extension, lateral bending and axial rotation. The parameters such as the RoM of each segment and the maximum stress of intervertebral disc were calculated. The purpose is to provide some guidance for selecting the height of cage during TLIF surgery for degenerative lumbar spine.

RoM is an indicator of spinal stability. If the segment stability is good after interbody fusion, the RoM is small [32]. On the contrary, if the RoM is large, the stability of fusion segment and adjacent segments after TLIF is poor. In the present study, as the degree of L3–L4 degeneration increased, the intervertebral space continuously decreased and osteophytes continuously enlarged, resulting in a negative amount of increase in RoM at this segment after L4–L5 implantation of the cage. With the increase in the height of the cage, the RoM of L3/L4 in the same degeneration group also increased, and the relationship between them was approximately linear (mild 1 mm/0.0179°, moderate 1 mm/0.0126°, severe 1 mm/0.0194°). The average increase of 14 mm was about 5.62% (mild), 3.83% (moderate) and 6.07% (severe) compared with 8-mm model.

The purpose of implanting cage is to open the appropriate intervertebral height and restore the normal physiological curvature of the lumbar spine. If the cage is too small, it cannot achieve the best treatment effect and even lead to the deformity of the fusion segment after operation, which will cause the patient's low back and leg pain and inconvenient walking [33]. It may also lead to insufficient decompression, the formation of pseudo-joints, increase the possibility of implant slippage, and increase the rate of surgical revision [27]. However, if the cage is too large, it may affect the recovery of spinal cord function [34], cause endplate damage [35], make the vertebral body bear greater load and increase the probability of cage sinking [36].

Due to the obvious reduction in intervertebral space and the small change of RoM of adjacent vertebral bodies when a larger cage is implanted, the mild-to-moderate degenerative spine can restore the normal lumbar curvature and avoid the significant loss of stability. Therefore, when TLIF is performed on mild-to-moderate degenerative lumbar spine, a cage slightly larger than the original space can be selected to achieve the purpose of optimal surgical treatment. For the lumbar spine with severe degeneration, although the intervertebral height can be restored when a larger cage is implanted, the RoM of adjacent segments may change greatly, which may cause spinal instability. Therefore, it is not recommended to implant a larger cage. The change of cage height had less effect on RoM of the lower adjacent segment in the mild-to-moderate group than that of the upper adjacent segment. In L5–S1 segment, the RoM of 14-mm model was about 4.44% (mild), 2.63% (moderate) and 19.36% (severe) higher than that of 8-mm model.

Biomechanics is one of the essential components in maintaining intervertebral disc homeostasis. For the mechanical load of intervertebral disc, physiological mechanical load is necessary to maintain the phenotype of intervertebral disc cells [37, 38], and excessive mechanical load may cause the obstacle of nutrient transport in the endplate, resulting in intervertebral disc injury and degeneration [39, 40]. Through finite element analysis, Du et al. [22] and Zhou et al. [29] found that both degenerative intervertebral disc and TLIF surgery increased the maximum stress of adjacent intervertebral disc, and in TLIF model, the increase in biomechanical parameters of adjacent surgical segments was more obvious than that in degenerative model.

In this study, with the increase in cage height, the maximum stress of L3–L4 intervertebral disc in the same degeneration group also increased correspondingly, and the relationship between them was approximately linear (mild 1 mm/0.0349 MPa, moderate 1 mm/0.04 MPa, severe 1 mm/0.0462 MPa). 14-mm model increased by about 10.89% (mild), 11.99% (moderate) and 13.64% (severe) compared with 8-mm model. The change of cage height had a greater impact on the maximum stress of intervertebral disc in the lower adjacent segment than that in the upper adjacent segment. In L5-S1 segment, the maximum stress of 14 mm model was about 27.00% (mild), 22.13% (moderate) and 29.84% (severe) higher than that of 8-mm model. For mild-to-moderate degenerative spine, implantation of larger cage during TLIF has less effect on the stress of adjacent intervertebral disc, and the aggravation of postoperative degeneration is not obvious. However, for the lumbar spine with severe degeneration, it is not recommended to implant a larger fusion cage to avoid aggravating the degeneration of adjacent segments.

There are some limitations in this study. Firstly, the health model came from only one volunteer, and we ignored the differences in different populations. Secondly, in order to simplify the model, the degeneration model was constructed only in L3–L4 segments. In fact, degeneration can occur in all segments. Finally, the TLIF operation only roughly simulated the real process, and the operation details could not be perfectly restored.

## Conclusion

In general, the finite element analysis showed that the RoM of adjacent segments and the maximum stress of intervertebral disc increased with the increase in cage height, and this trend was not obvious in mild-to-moderate degeneration models. For patients with mild-to-moderate lumbar degeneration requiring TLIF surgery, a cage no more than 2 mm larger than the original intervertebral space can restore the normal lumbar disc height to the greatest extent and reduce the increase in RoM and stress in adjacent segments as much as possible, so as to achieve the best treatment effect without accelerating the degeneration of adjacent segments. However, for patients with severe lumbar degeneration, it is better to select a fusion cage close to the original intervertebral height, and the intervertebral space should not be overstretched.

## Data Availability

The data sets used and analysed during the current study are available from the corresponding authors on reasonable request.

## Abbreviations

TLIF: Transforaminal lumbar interbody fusion
RoM: Range of motion
ASD: Adjacent segment degeneration
